# Probabilistic electrical stimulation mapping of human medial frontal cortex

**DOI:** 10.1016/j.cortex.2018.06.015

**Published:** 2018-12

**Authors:** Gianluca Trevisi, Simon B. Eickhoff, Fahmida Chowdhury, Ashwani Jha, Roman Rodionov, Mark Nowell, Anna Miserocchi, Andrew W. McEvoy, Parashkev Nachev, Beate Diehl

**Affiliations:** a Neurosurgery Department, Fondazione Policlinico Universitario A. Gemelli, Catholic University Medical School, Rome, Italy; b Institute of Systems Neuroscience, Medical Faculty, Heinrich Heine University Düsseldorf, Düsseldorf, Germany; c Institute of Neuroscience and Medicine, Brain & Behaviour (INM-7), Research Centre Jülich, Jülich, Germany; d National Hospital for Neurology and Neurosurgery, London, UK; e Institute of Neurology, UCL, London, UK

**Keywords:** Direct electrical cortical stimulation, Supplementary motor cortex, Epilepsy surgery, Intracranial EEG recordings, Probability estimate

## Abstract

The medial frontal cortex remains functionally ill-understood; this is reflected by the heterogeneity of behavioural outcomes following damage to the region. We aim to use the rich information provided by extraoperative direct electrical cortical stimulation to enhance our understanding of its functional anatomy.

Examining a cohort of 38 epilepsy patients undergoing direct electrical cortical stimulation in the context of presurgical evaluation, we reviewed stimulation findings and classified them in a behavioural framework (positive motor, negative motor, somatosensory, speech disturbances, and “other”). The spatially discrete cortical stimulation-derived data points were then transformed into continuous probabilistic maps, thereby enabling the voxel-wise spatial inference widely used in the analysis of functional and structural imaging data.

A functional map of stimulation findings of the medial wall emerged. Positive motor responses occurred in 141 stimulations (31.2%), anatomically located on the paracentral lobule (threshold at *p*<.05), extending no further than the vertical anterior commissure (VCA) line. Thirty negative motor responses were observed (6.6%), localised to the VCA line (at *p* < .001 uncorrected). In 43 stimulations (9.5%) a somatosensory response localised to the caudal cingulate zone (at *p* < .001 uncorrected), with a second region posterior to central sulcus. Speech disturbances were elicited in 38 stimulations (8.4%), more commonly but not exclusively from the language fMRI dominant side, just anterior to VCA (*p* < .001 uncorrected). In only 2 stimulations, the patient experienced a subjective “urge” to move in the absence of overt movement.

Classifying motor behaviour along the dimensions of effector, and movement *vs* arrest, we derive a wholly data-driven stimulation map of the medial wall, powered by the largest number of stimulations of the region reported (*n* = 452) in patients imaged with MRI. This model and the underlying data provide a robust framework for understanding the architecture of the region through the joint analysis of disruptive and correlative anatomical maps.

## 1 Introduction

The human medial frontal cortex participates in a great number of motor, behavioural and cognitive tasks as shown in clinical and experimental correlative studies (Filevich et al., 2012, Luders et al., 1995, Nachev et al., 2008, Penfield, 1950, Zilles et al., 1995). The remarkable prevalence of functional imaging activation here has generated a wide diversity of attributed roles, but a cohesive, synoptic theory of its contribution to thought and behaviour is yet to emerge. A key factor in this lack of definition is the comparative rarity of data from disruptive methods—pathological or experimental—that can robustly test the *necessity* of the neural substrate for any putative role. Stroke, the commonest natural cause of focal brain damage, rarely affects the medial wall (Mah, Husain, Rees, & Nachev, 2014), and perhaps the best established experimental disruptive technique, repetitive transcranial magnetic stimulation, cannot easily reach its depths without potentially confounding effects from the surface.

Direct electrical cortical stimulation in the context of surgical planning has arguably the greatest power to illuminate the function of the region in humans. Although *intraoperative* stimulation offers excellent flexibility of localisation, access interhemispherically remains technically challenging, and the intraoperative context limits the range of testable behaviours. *Extraoperative* cortical stimulation during chronic intracranial EEG investigations for presurgical diagnostics in patients with refractory epilepsy permits the evaluation of behaviours of arbitrary complexity (Lim et al., 1994). Electrodes are placed on the medial frontal wall for localisation of the epileptogenicity and for cortical mapping, according to the clinical hypothesis. The epilepsy may be suspected to arise in or close to the supplementary motor area (SMA) necessitating close coverage of this area, or seizures may involve the medial frontal wall during their evolution. Coverage of the area can be achieved with good sampling density from grid electrodes placed interhemispherically, or during a Stereo EEG exploration (SEEG) by inserting one or several depth electrodes targeting various locations along the medial wall. Precise knowledge regarding the location of various functionalities of the medial frontal wall are hence necessary for successful implantations. Focal epilepsies arising from the medial frontal wall often respond favourably to surgical resection (Alonso-Vanegas et al., 2017) and counselling regarding risks for a deficit is informed by cortical mapping and the planned resection margin.

The highly individualised electrode placement, necessarily dictated by clinical need, makes population-level anatomical inference difficult. We therefore need new methodology to integrate sparsely sampled information across individuals, enabling population-level functional-anatomical inference, both to illuminate the role of the region in behaviour and to guide surgical resection boundaries. This task requires a means of modelling the anatomical correspondences across different individuals where the sampling in each is discrete, sparse and variable as necessitated by clinical placement of electrodes. The established approach is to classify behavioural responses within anatomically predefined, region-of-interest parcellations of the brain. But such an approach *presupposes* the functional-anatomical boundaries rather than materially contributing to their definition (Eickhoff, Constable, & Yeo, 2017). To infer them *from the data itself*, here we develop a solution drawn from the field of imaging meta-analytics, where spatially continuous, probabilistic inferences are analogously derived from discrete point-coordinate data (Eickhoff et al., 2012, Eickhoff et al., 2009). The key premise of our approach is that inter-individual variations in localisation are reasonably modelled by a random Gaussian field. Such an approach is well-established in volumetric brain morphometry and functional imaging (Friston et al., 1995), and more conservative and less potentially biasing than the assumptions underlying any region-of-interest parcellation.

Our objective is to enhance our knowledge of the functional anatomy of the human medial wall. Current functional maps are derived either from purely correlative data such as fMRI or electrical stimulation data analysed within landmark-defined regions-of-interest that *presuppose* an underlying functional-anatomical organization rather than infer it from the data (Chauvel et al., 1996, Lim et al., 1994). This allows for the first time to derive a wholly data-driven, continuous electrical stimulation map of the medial frontal wall. In 38 patients with drug-resistant epilepsy undergoing intracranial EEG recordings at the National Hospital for Neurology and Neurosurgery, we categorised behaviour in response to electrical stimulation of the 538 implantation sites into 5 standard groups. We then used the spatial information of electrode locations to reveal a new topography of the medial wall. This not only sheds light on the fundamental organisation of the region, but also constitutes a useful empirical prior for guiding individual electrode placement in a clinical context.

## 2 Materials and methods

### 2.1 Study population

We reviewed clinical data from 147 consecutive patients with drug-resistant focal epilepsy who underwent prolonged intracranial recording at the National Hospital for Neurology and Neurosurgery, London as part of clinical care, between January 2008 and June 2015. The study was approved by the hospital as retrospective review.

Thirty-eight patients (29 male, 9 female, aged 18–50 years age, mean age or 32.4 with a standard deviation of 10.3 years) were identified to have had at least one stimulated electrode in the medial frontal region, defined as the area on the medial wall rostral to the caudal bank of the marginal sulcus and dorsal to the corpus callosum. Table 1 summarises the patient demographic and clinical characteristics. Although five patients had small lesions on MRI in the vicinity of the SMA, only one had a lesion in the SMA/paracentral lobule. Further clinical details are given in Supplementary Table1.

**Table 1:** Demographic and clinical characteristics.

Feature	Value
*Demographics*	
Females	9 (24%)
Males	29 (76%)
Mean age at study	32.4 years (sd = 10.3)
*Clinical*	
Mean age of epilepsy onset	11.8 years (sd = 9.2)
Mean duration of epilepsy	22 years (sd = 10.1)
*Type of study*	
Grid electrodes	19 (50%)
Grid & depth electrodes	13 (34%)
SEEG	19 (50%)
*Side of study*	
Dominant	19 (50%)
Non-dominant	18 (47%)
Bilateral	1 (3%)
*Epileptogenic zone involvement*	
Frontal lobe	29 (76%)
Medial frontal wall	19 (50%)
*Abnormal MR imaging*	
Frontal lobe	10 (26%)
On or near medial wall	5 (13%)
*Medial wall resection done or planned*	14 (37%)

### 2.2 Electrode implantation

Nineteen patients (16 males, 3 females) underwent the implantation of depth electrodes with a previously described frameless-SEEG technique (Nowell et al., 2014). Nineteen patients (13 males, 6 females) underwent a craniotomy and insertion of grids and/or strips with or without navigation-guided free hand insertion of additional depth electrodes. Side of intracranial recording was left hemisphere in 21 cases (9 SEEG, 12 Grids/Strips), right in 16 (9 SEEG, 7 Grids/Strips) and bilateral in 1 (SEEG). According to language dominance determined by fMRI, in 19 patients (50%) the recording electrodes were located in the dominant hemisphere, in 18 (47%) in the non-dominant hemisphere, and were bilateral in one (3%). All patients underwent a post-implantation CT or MRI scan to confirm electrode location.

### 2.3 Direct electrical stimulation

An experienced epileptologist and a physiologist performed one or more sessions of cortical stimulation under continuous Video-EEG recording, typically after completion of ictal recordings and when on baseline antiepileptic medications, as part of the clinical investigations. Stimulation was bipolar or monopolar, delivering trains of bi-phasic square wave pulses of AC-current at 50 Hz, pulse width 500 μs, with a maximum duration of 5 s. The stimulation intensity was increased in a stepwise fashion from .5 up to 7 mA in increments of .5–1 mA or until the occurrence of a clinical sign or after-discharges on EEG monitoring (Kovac et al., 2014).

Stimulation testing was performed independently at rest, during Barré and/or Mingazzini test, finger tapping and/or alternate movements of supination and pronation of the forearm or of dorsal and plantar flexion of the ankle, and during counting, reading, or repetitive monosyllabic verbalisation. Patients were asked to describe any perceived motor, sensory or cognitive phenomena after each period of stimulation, both at rest and during any of the above tasks.

### 2.4 Behavioural analysis

Behavioural responses were classified by three assessors (GT, FC & BD) as positive motor, negative motor, somatosensory, speech disturbances, and “other” for those, such as urges, which did not fall into any of the preceding categories. Classification was performed live by the stimulating and attending clinicians as part of the clinical record, and confirmed *post hoc* from video and audio telemetry data sourced from two camera angles. Where no responses were obtained at stimulations up to 7 mA the contact was considered to be silent. Responses followed by seizures or after-discharges were excluded from the analysis.

Positive motor responses constituted overt, involuntary movements of the eyes, head, limbs or trunk, either tonic or clonic, subdivided by somatotopy. Negative motor responses constituted slowing or inhibition of the tested active movements, including inability to maintain a sustained position during the Barré or Mingazzini test. The observation of a positive response at rest and a negative response during a task was labelled as a “complex” response. Somatosensory responses, somatotopically labelled, constituted such perceptions as the patient reported on general prompting: these ranged across cutaneous paraesthesias of tingling, touch, heat, or pain. Speech disturbances included arrest, hesitation, change in speech rhythm (slowing or acceleration), and involuntary vocalisation. The category of “other” included phenomenology falling outside the preceding categories: the urge to move or speak without actual movement or verbal output, and the perception of visual motion.

### 2.5 Imaging data

Pre-operative structural imaging consisted of a whole-brain, T1-weighted magnetic resonance scan, typically of ∼1 mm isotropic resolution, and acquired on a 3T scanner. Post-implantation structural imaging consisted of an uncontrasted, whole-head, CT scan of resolution .43 × 0.43 × 1.2 mm (SOMATOM Definition 128-slice, Siemens Healthcare GmbH, Erlangen, Germany).

Functional MRI data were acquired on a 3T General Electric Excite HDx scanner (General Electric, Milwaukee, WI, USA) as gradient-echo planar T2*-weighted images (TE = 30 ms, TR = 4.5s) providing blood oxygenation level dependent (BOLD) contrast. Each volume comprised 58 contiguous 2.5 mm oblique axial slices, through the temporal and frontal lobes with a 24 cm field of view, 96 × 96 matrix, reconstructed to 128 × 128 for an in-plane resolution of 1.88 × 1.88 mm.

#### 2.5.1 Structural image processing

To allow a population-level spatial inference, individual electrode contact locations were non-linearly transformed into a common Montreal Neurological Institute (MNI) space (Jha, Diehl, Scott, McEvoy, & Nachev, 2016). All image processing was carried out within SPM12 (http://www.fil.ion.ucl.ac.uk/spm/). For both the pre-operative T1 and the post-implantation CT of each patient, a rigid body co-registration to the standard SPM12 tissue probability map was first performed based on normalised mutual information with adjustment from a Procrustes analysis weighted by the white and grey matter compartments. This placed each scan in approximate rigid register with the MNI template space, making subsequent transformations more robust. The CT scan was then co-registered to the T1 with SPM12's standard co-registration routine (spm_coreg.m), allowing subsequent operations on the T1 to be directly replicable on the CT. SPM12's standard unified segmentation and normalisation procedure (spm_preproc.m), with default parameters, was then applied to the T1 to generate segmented compartments in native space for each of the standard 6 tissue classes, as well as a set of parameters for non-linear transformation into MNI space of this and any other image in register with it. These parameters were used to transform the grey and white matter compartments of each T1 scan—and the corresponding CT—into normalised MNI space. To localise the electrode contacts in MNI space, each set of normalised T1 and CT scans was displayed in linked, triplanar view (using SPM's “check registration” module), and the location of the centre of contacts visually judged to fall within the grey matter of the medial wall was manually noted by two observers. Electrode localisation was performed in MNI space rather than native space prior to normalisation so as to minimize the potentially biasing effects of inter-subject anatomical differences in the manual labelling process. For display purposes, the normalised grey and white matter compartments of all patients were independently averaged to create a single group template.

#### 2.5.2 Functional image processing

Two tasks were performed in separate imaging sessions. The “verb generation” task consisted of eight blocks lasting 30 s each, interspersed with periods of rest fixation of the same length, where the patients were visually presented with concrete nouns every 3s, and were asked either covertly to generate verbs associated with these nouns (indicated by the letter “G” preceding the noun), or silently to repeat the nouns presented (indicated by the letter “R” preceding the noun). Each block contained trials of the same type, with the verb generation and noun repetition alternating across blocks. The “verbal fluency” task consisted of a blocked experimental design with 30 sec activation blocks alternating with 30 sec of cross-hair fixation during the baseline condition over 5.5 min. During the activation phase, subjects were asked to covertly generate different words beginning with a visually presented letter (A, S, W, D and E).

The fMRI time-series of each subject was realigned using the mean image as a reference, and smoothed with a Gaussian kernel of 8 mm full-width at half maximum. At each voxel, the time-series BOLD data were entered into a general linear model with the conditions modelled as a box-car function regressors convolved with a standard haemodynamic response function. The realignment parameters were added as nuisance regressors to minimize artefactual activation owing to head movement correlated with the task. Main effects were estimated separately for each condition. For the “verb generation” task, the contrast of interest was between blocks of noun repetition and verb generation. For the “verbal fluency” task, between blocks of word generation and fixation. The resultant contrast images were normalised into Montreal Neurological Institute (MNI) stereotactic space by applying the necessary non-linear deformation field estimated from the pre-operative T1 MR scan after co-registration to the mean image of the fMRI time series. Separately for each task, these normalised contrast images were entered into a second level voxel-wise analysis consisting of a one-sample *t* test, thresholded at *p* < .05 family wise error corrected.

### 2.6 Probabilistic stimulation mapping

All sampled electrode contact locations were reflected onto one hemisphere and then modelled as 3D Gaussians centred on the irrespective locations, analogously to the approach taken in meta-analytic modelling of imaging activation data (Eickhoff et al., 2009, Eickhoff et al., 2012). This manoeuvre allows us to capture inter-subject variations in functional–anatomical relationships incompletely eliminated by anatomical registration, and provides whole volume support for each data point, enabling the application of voxel-wise mass-univariate statistics for spatial inference.

Based on previous empirical data on spatial uncertainty of functional neuroanatomy across subjects (Eickhoff et al., 2009), we primarily employed a kernel of 10 mm FWHM (truncated at 90% mass). Kernel widths from 4 to 16 mm in 2 mm steps were additionally evaluated and yielded closely comparable results, confirming that the choice of kernel size is not a critical step.

In line with other analyses of the medial wall, we removed the effect of lateralisation in order to focus on the key question of the rostrocaudal organisation (Nachev et al., 2008). Owing to the overwhelming proportion of right handers (34 of 38) and left hemisphere language dominants (29 of 38, the rest being bilateral) we do not have the statistical power to probe hemispheric effects. Given our focus on the rostrocaudal anatomy here it is appropriate to collapse data across hemispheres to maximise the yield of the anatomical contrast of interest.

The analysis volume was defined to encompass the medial frontal wall, extending laterally 22 mm to enclose the main peak of sampling density. For each 2 × 2 × 2 mm voxel of the analysis volume we then derived condition-specific, voxel-wise, response probability maps by computing the activation likelihood estimation (ALE) score (Eickhoff et al., 2009, Eickhoff et al., 2012) across all recordings yielding the respective behaviour. The ALE score is simply the union of activation probabilities for each voxel corresponding to the modelled behaviour, a measure of the association of each voxel with the behaviour, exactly as in the meta-analytic case from which the approach is derived. Of course, here instead of activation we have Gaussian smoothed points of stimulation, but the spatial inference remains the same.

In this case the *proportion* of stimulations, rather than the total number, that resulted in a specific behavioural effect is most meaningful especially because the total number of stimulations varies across the medial wall. The *absolute* condition-specific ALE-score maps were therefore converted into *relative* maps, by normalising with respect to an ALE-score map of the total number of stimulations across all behavioural conditions. This later map–generated by convolving *all* stimulation locations with a Gaussian kernel–can be interpreted as a “stimulation sampling density” map, where the value at each voxel reflects the likelihood of stimulation testing there. Permutation testing over 25000 iterations under the null hypothesis of exchangeability of behavioural categories (analogous to the contrast approach in ALE analyses, cf. Eickhoff et al., (2011) was then used to derive voxel-wise *z* score maps that reflect the voxelwise association between stimulation and each specific (rather than a random) behavioural effect. These behaviour-specific images were thresholded at the levels of *p*<.001 uncorrected and *p*<.05 family-wise error corrected. Since our specific interest is in the organisation in the rostro-caudal plane, the maps are show as maximum intensity projections through the region of interest.

## 3 Results

**Fig. 1:**
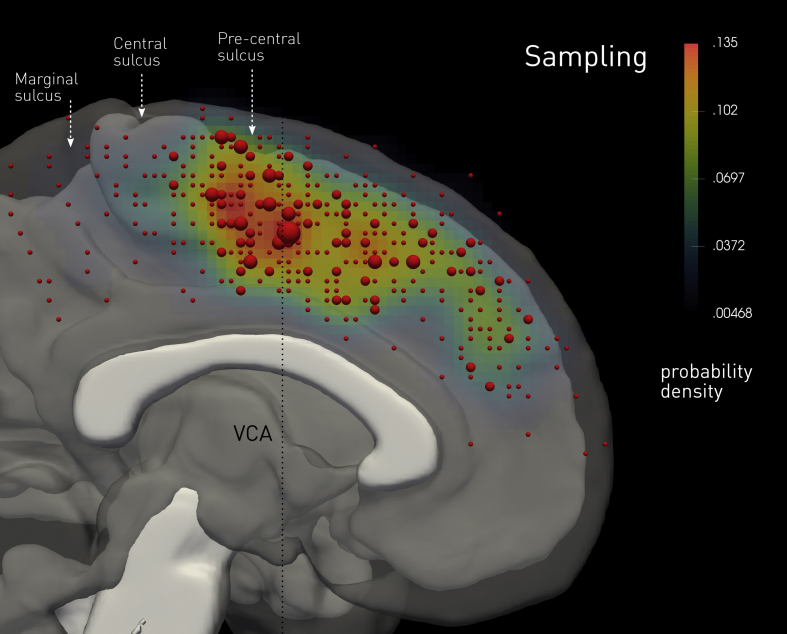
**Distribution of stimulation locations across the medal wall.** The red spherical glyphs show the stimulated locations in MNI space in the rostrocaudal plane, where sampled more than once indicated by a proportionately larger diameter glyph. The colourmap shows the continuous probability density of sampling in the same plane, derived by convolving each stimulated location with a Gaussian kernel of 10 mm full-width-at-half-maximum. The locations are summed in the coronal plane extending 22 mm from the midline and transposed to one hemisphere. The image underlay is composed of the median of the normalized white matter tissue probability maps of all participants, thresholded at *p* < .05 (in white), and the median of the normalized grey matter tissue probability maps of all participants, thresholded at *p* < .05 (in translucent grey).

**Fig. 2:**
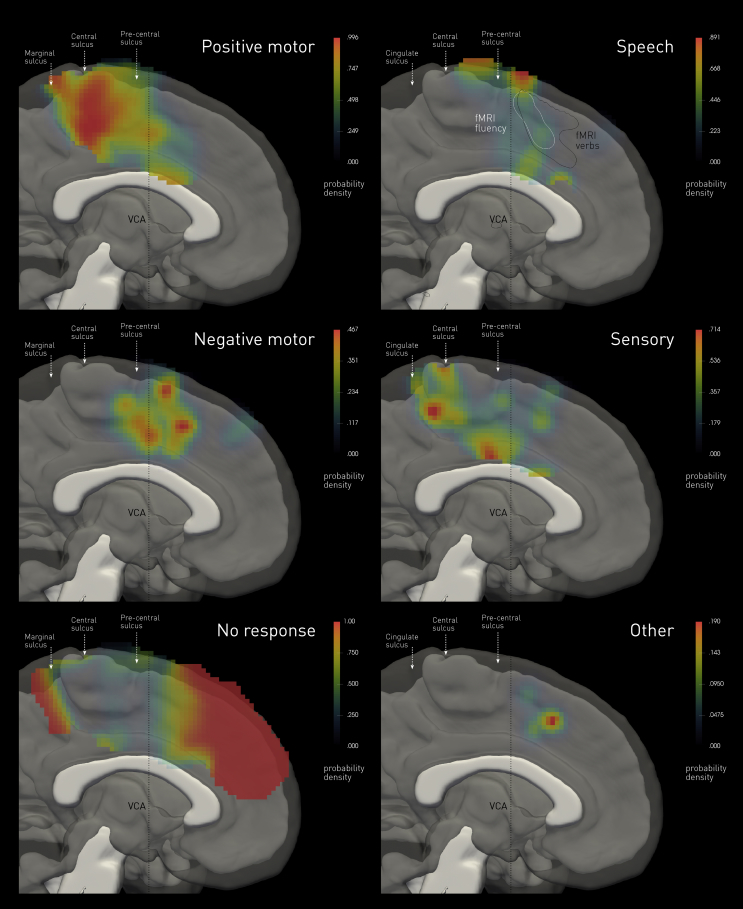
**Probability density maps of stimulation-induced behaviours.** The colourmaps show the estimated probability density of eliciting each of six categories of behaviour, including no response at all, derived by convolving each stimulated location where the behaviour is observed with a Gaussian kernel of 10 mm full-width-at-half-maximum, normalised by the overall sampling density. The white and black contours labelled “fMRI” show the *p* < .05 family-wise error corrected boundaries in the rostrocaudal plane of BOLD activation observed in the fluency and verb generation tasks across the group. The image underlay is composed of the median of the normalized white matter tissue probability maps of all participants, thresholded at *p* < .05 (in white), and the median of the normalized grey matter tissue probability maps of all participants, thresholded at *p* < .05 (in translucent grey).

**Fig. 3:**
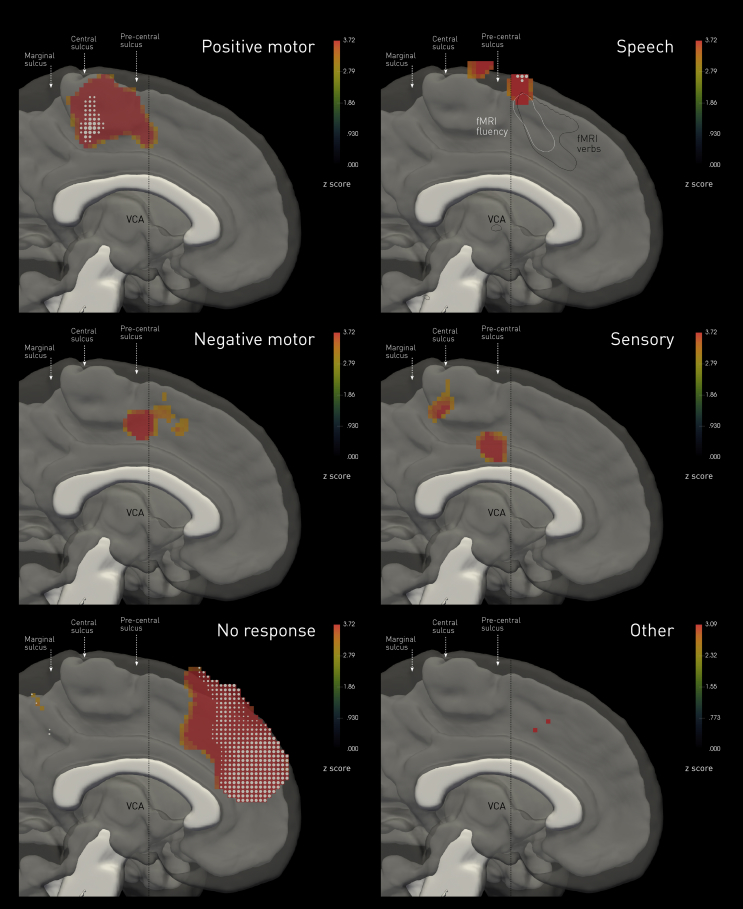
**Permutation-based voxel-wise statistical maps of stimulation-induced behaviours.** The colourmaps show the estimated voxel-wise *z* score for a permutation test of the null hypothesis of exchangeability of behavioural categories in response to stimulation. The colourmaps are thresholded at *p* < .001 uncorrected. Voxels labelled with white glyphs indicate those surviving a threshold of *p* < .05 family-wise error corrected. The white and black contours labelled “fMRI” show the *p* < .05 family-wise error corrected boundaries in the rostrocaudal plane of BOLD activation observed in the fluency and verb generation tasks across the group. The image underlay is composed of the median of the normalized white matter tissue probability maps of all participants, thresholded at .05 (in white), and the median of the normalized grey matter tissue probability maps of all participants, thresholded at .05 (in translucent gray).

A total of 452 stimulations across 538 contacts were obtained as depicted in Fig. 1. The overall distribution of sampling showed greatest density in the vicinity of the vertical anterior commissure (VCA) line. The spatial distribution of each stimulation-induced behavioural category, normalised by the overall sampling density, is given in Fig. 2. Formal statistical maps of the functional anatomy are given in Fig. 3. These show the outcome of a voxel-wise permutation test of the null hypothesis of exchangeability of behavioural categories, i.e., the extent to which the voxel density associated with the specified category may be expected to have arisen by chance. Each figure also shows the localisation on the medial wall of standard language tasks used in hemispheric dominance testing. A summary of the catalogued behaviours is given in Table 2.

**Table 2:** Behavioural responses to cortical stimulation of the medial frontal wall.

Response	Total Responses (mA)	Patients	Simple (mA)	Complex (mA)
Positive Motor	133 Tonic (2.33 ± 1.12) 8 Clonic (2.12 ± 1.12)	30	110 (2.28 ± 1.09)	9 SSR (2.5 ± .93) 8 NMR (3.19 ± 1.93) 6 NMR + SD (2.33 ± .68) 5 SD (2.7 ± .45) 2 NMR + SSR (2) 1 NMR + SSR + SD (3)
Negative Motor	30 (3.03 ± 1.38)	19	5 (3.1 ± .55)	8 PMR (3.19 ± 1.93) 7 SD (3.71 ± 1.7) 6 PMR + SD (2.33 ± .68) 2 PMR + SSR (2) 1 SSR + SD (3) 1 PMR + SSR + SD (3)
Somatosensory	43 (2.07 ± .8)	18	29 (1.8 ± .7)	9 PMR (2.5 ± .93) 2 PMR + NMR (2) 1 PMR + NMR + SD (3) 1 NMR + SD (3) 1 SD (3)
Speech Disturbance	38 (2.84 ± 1.08)	15	17 (2.68 ± .98)	7 NMR (3.71 ± 1.7) 6 PMR + NMR (2.33 ± .68) 5 PMR (2.7 ± .45) 1 SSR (3) 1 NMR + SSR (3) 1 PMR + NMR + SSR (3)al
Urgencies	2 (1.5 ± .5)	2	2 (1.5 ± .5)	–

Stimulation sometimes induced a combination of two or more behaviours, labelled a complex response. The numbers in brackets indicate the average amplitude of stimulation in mA ± standard deviation. A frequency of 50 Hz and pulse width of 500 μs were used in all stimulations. Abbreviations: NMR, negative motor response; PMR, positive motor response; SD, speech disturbance; SSR, somatosensory response.

### 3.1 Positive motor responses

These were the commonest overt responses, occurring in 141 stimulations (31.2% of total). The majority (131) were tonic, involving the entire limb with a proximal emphasis, almost always contralateral to stimulation, and rarely bilateral. Where clonic (8), they were always contralateral to the stimulation side. Positive motor responses were the only inducible response in 110 stimulations; in the remaining 31, at least one other kind was observed, yielding a “complex” response. Anatomically, the responsive areas covered occupied the paracentral lobule, surviving the significance threshold of *p*<.05 corrected within the caudal segment, bordering inferiorly on the cingulate sulcus, and extending no further than the VCA line (Fig. 2, Fig. 3).

### 3.2 Negative motor responses

Such responses were observed on 30 occasions (6.6% of all stimulations), where isolated localising to the upper limbs in four cases (one bilateral) and to one foot in one case. Where complicated by at least one other category of response (25), they were associated with positive motor (17) or speech effects (15), generally contralateral or less frequently bilateral. Anatomically, negative motor responses localised to the VCA (at *p* < .001 uncorrected), corresponding to the hypothesized junction between the supplementary and presupplementary motor areas (Fig. 2, Fig. 3).

### 3.3 Somatosensory responses

In 43 stimulations (9.5% of total) a somatosensory response was observed, the only elicitable response in a third of these. Qualitatively, they were paraesthesias (19), dysesthesias (13), pain (6), twitching sensation (3), altered spatial awareness (1), and a warm sensation (1). Anatomically, sensory responses localisedto the caudal cingulate zone (at *p* < .001 uncorrected), in keeping with the known greater sensory responsiveness of the region in comparison with other areas on the medial frontal lobe, and within a distinct second region just posterior to the central sulcus (Fig. 2, Fig. 3).

### 3.4 Speech disturbance

Speech arrest, hesitation or prosodic slowing of speech, or vocalisations was elicited in 38 stimulations (8.4% of total). Stimulations of the fMRI-determined dominant hemisphere more commonly, but not exclusively, evoked such responses. Anatomically, the responsive area (at *p* < .001 uncorrected), overlapped with only the most dorsocaudal margin of the areas activated during language fMRI tasks on the medial wall, just anterior to the VCA. The behavioural correlates in this cluster (seen in 17/38 stimulations) typically were not associated with positive motor phenomena; negative motor behaviour of the tongue was however only excluded in four patients. A further cluster, falling within the presumed supplementary motor area, survived the threshold of *p* < .001 uncorrected only (Fig. 2, Fig. 3), and those were commonly complicated by motor responses of head/face/tongue, or the upper limbs.

### 3.5 Other

In only 2 of all 452 stimulations, the patient experienced a subjective “urge” to move in the absence of any overt movement. On one occasion a patient reported a sense of visual motion. Anatomically, this area was deeply rostral of the VCA, though so few responses constitute weak grounds for localisation (Fig. 2, Fig. 3). We draw attention to this category only because so much is made of it in the voluntary action literature.

### 3.6 No response

The commonest effect of stimulation of the medial wall was no objective or subjective behavioural response (250 stimulations, 55.3% of total). Anatomically, this covered an extensive area well anterior to the VCA, overlapping caudally with the rostral component of the fMRI landmark for the presupplementary motor area (Fig. 2, Fig. 3). The caudal bank of the marginal sulcus was also silent. The anterior component of this area survived the significance threshold of *p*<.05 family wise error corrected.

## 4 Discussion

Though necessarily constrained by the demands of clinical practice, this is to our knowledge the largest and most comprehensive evaluation of the behavioural effects of direct electrical stimulation of the human medial frontal wall in the MRI era, and the first using methodology allowing for probabilistic statistical inferences by generating permutation-based voxel-wise statistical maps of stimulation-induced behaviours. It reveals several features of neuroscientific and clinical translational significance.

In striking contrast to the remarkable eloquence of the region in functional imaging studies, the commonest effect of stimulation here was no objective or subjective response, especially rostral to the VCA line. This suggests the activity of the region must be strongly task-dependent, across an array of behaviours that are currently untested in routine clinical practice. If direct cortical stimulation is to illuminate the function of the region—and to aid in the delineation of resection margins—we must clearly expand the evaluated behavioural repertoire, inevitably in the direction of greater complexity. This is particularly true given the frequent co-activation of the medial wall during a wide spectrum of behavioural tasks (de la Vega, Chang, Banich, Wager, & Yarkoni, 2016).

Task specific paradigms to be applied during perturbation using cortical stimulation will require to be very short (ideally 5 sec, as is customary in clinical practice). Limitations of functional delineation using cortical stimulation include the concern of current spread beyond the stimulated site. It is therefore critical that the paradigms are performed at the lowest possible stimulation current intensity to avoid excessive spread (Winawer & Parvizi, 2016), with continued ECoG monitoring to recognise afterdischarges or seizures. If meaningful conclusions for both scientific insights and patient care should be derived, a systematic test battery needs to be devised, that is accessible to patients with various levels of cognitive difficulties and may be customisable to their abilities, as determined in a testing session outside the stimulation session. This provides an opportunity to engage with the wider neuroscientific community to develop such batteries, which then need to be prospectively validated.

Our map compels a re-examination of the anatomy of the area in several important respects.

First, we demonstrate that the functionally-defined SMA extends caudally as far as the inferior aspect of the paracentral lobule, further than the widely accepted caudal boundary. This has been previously suggested (Lim, Dinner, & Luders, 1996) but not confirmed and is at odds with the most widely held neurosurgical definition. The surgical perspective is based on deficits observed following resections of the presumed SMA, which typically do not include primary leg and foot motor cortex, due to the permanent deficit such a resection would induce (Fontaine et al., 2002, Zentner et al., 1996), and only involves medial cortex anterior to paracentral lobule. It is noted that such lesioning typically leads to a characteristic typically transient deficit showing much less involvement of lower compared to upper extremity. In this study, we were able to transiently disrupt the paracentral lobule with extraoperative cortical stimulation, and propose a different functional anatomical boundary. We elicited predominantly tonic limb movements, often proximal in nature as is characteristic of SMA type motor response (Lim et al., 1996) from the paracentral lobule. This lends support to a previous stimulation study approximating anatomic relation of stimulation findings using lateral skull x-rays to identify electrode positions and superimposing them on preoperative MRIs, revealing an approximation of a functional map (Lim et al., 1994).

Second, we reveal a complex co-localisation of positive and negative stimulation induced motor responses, far from the macroscopic division between generative and inhibitory areas widely assumed to map across the SMA/preSMA boundary. This is especially important given the difficulty of mapping putatively inhibitory functions with fMRI, and the importance of preserving negative motor areas during resective surgery to reduce the incidence of supplementary motor area syndrome and bimanual co-ordination deficits (Rech et al., 2014, Schucht et al., 2013).

Third, we show a marked discrepancy between the areas that show language-related activity on fMRI and the areas whose stimulation disrupts language production.

The nature of the speech disturbances elicited using cortical stimulation of the medial frontal wall, first reported by Penfield and Welch (Penfield & Welch, 1951) and again demonstrated in this study, has been widely debated (Chauvel et al., 1996, Lim et al., 1996). Speech disturbance can be caused by positive or negative motor phenomena of the mouth or tongue, and we found such an association with tongue or mouth positive motor responses in several cases, typically elicited from a location caudal to the VCA line. In others, no overt motor phenomenon was reported and those cases mapped to a location superiorly and just anterior to the VCA line. A limitation of the retrospective clinical nature of the study is that whilst positive motor associated responses were always documented, inability for tongue movement was only tested in some of the cases, and then absent in half. Although numbers are small, our findings support two areas form which speech production was disrupted, supporting the concept of a parallel structure, i.e., a link of SMA proper to the articulatory-phonological network whereas pre-SMA is predominantly linked to Broca's area (Hertrich, Dietrich, & Ackermann, 2016).

Fourth, comprehensive coverage of the medial wall across a wide range of stimulated intensities yielded a reported “urge” on only 2 of 452 possible occasions. Such phenomenology, described by Fried et al., (1991), has been widely used to support intentionalist models of voluntary action (Libet, 1985), despite being rare even in the original description (15 of 129 stimulated sites). Some of the most highly—and approvingly—cited papers in the literature on voluntary action are founded on such models, and here their criticism on conceptual grounds (Nachev, 2011, Rothwell and Edwards, 2011) is given strong empirical support.

The discrepancy between fMRI and stimulation maps shows neither renders the other technique redundant; rather, we must consider how best to combine the two modalities within the same inferential setting (Eickhoff et al., 2017). We have offered a principled method for achieving this, transforming stimulation data into the familiar statistical framework of functional imaging and volumetric brain morphometry.

Finally, we show that the framework of meta-analytic neuroimaging is readily adapted to the analysis of direct electrical stimulation, enabling the principled derivation of continuous functional maps from sparse, discretely sampled data. Though implemented here with highly conservative, unimodal mass-univariate statistics, the approach is readily extensible to other forms of spatial inference, including jointly with data from other investigative modalities.

The focus of the present analysis is the rostro-caudal organisation of the medial wall, motivating us to collapse across other dimensions so as to maximise our power in the anatomical plane of interest. The approach may nonetheless be applied with no constraint, as the functional anatomical or clinical question in hand requires, for stimulation points are modelled fully volumetrically.

These data and the novel methodology introduced to model them provide a robust framework for understanding the architecture of the region and its differential appearance to disruptive and correlative mapping. Using this framework, carefully designed prospective studies correlating stimulation findings in response to complex behavioural testing will allow to obtain a much more detailed map of the medial wall.
