# Supplementary material for: Probabilistic electrical stimulation mapping of human medial frontal cortex
Source: Cortex. 2018 Dec;109:336–46. doi: 10.1016/j.cortex.2018.06.015 (PMC6259584; doi:10.1016/j.cortex.2018.06.015)
Supplement: Multimedia component 1 [file mmc1.pdf]

Supplementary Table 1: Patient clinical characteristics

Patient	Type	Sex	Handed	Age at onset	Age at ICR	Epilepsy duration	Side	Language dominance	Imaging abnormality	Ictal onset zone	Pathology, if operated	ILAE outcome (months)
1	Grid	F	Left	3	27	24	Left	Left	None	Left SFG	FCD iia	1 (39)
2	Grid	F	Right	12	36	24	Left	Left	None	Left mesial SFG	Non-specific	5 (54)
3	Grid	M	Right	6	26	20	Right	Bilateral	None	Right SFG/precentral	FCD iia	4 (33)
4	Grid	F	Right	10	28	18	Right	Left	None	Right SFG	FCD iib	1 (54)
5	Grid	F	Right	8	23	15	Left	Left	Left insula lesion	Left anterior frontal	Gliosis	5 (27)
6	Grid	M	Right	12	20	8	Left	Left	None	Left pre/post central/SMA	Inoperable	N/a
7	Grid+depth	M	Right	16	45	29	Left	Left	None	Not localised	Inoperable	N/a
8	Grid+depth	M	Right	27	40	13	Right	Left	None	Right SFG	FCD iib	1 (60)
9	Grid+depth	M	Right	2	21	19	Right	Left	Dysplastic left precentral gyrus	Primary motor area	Inoperable	N/a
10	Grid+depth	M	Right	38	48	10	Left	Left	None	Left SMA	Inoperable	N/a
11	Grid+depth	M	Right	10	23	13	Right	N/a	None	Not localised	Inoperable	N/a
12	Grid+depth	M	Right	6	25	19	Left	Left	Left central gyrus signal change	Right hand sensory motor area	Inoperable	N/a
13	Grid+depth	F	Right	11	21	10	Left	Left	Cerebellar lesion	Left primary motor cortex	Inoperable	N/a
14	Grid+depth	M	Right	8	18	10	Left	Left	Left precentral gyrus tumour	Left paracentral/precentral	Inoperable	N/a
15	Grid+depth	M	Right	6	38	32	Right	Left	Dysplastic right SFG	Right SMA	Gliosis	5 (42)
16	Grid+depth	M	Right	29	41	12	Left	Left	None	Left SMA	Non-specific	5 (12)
17	Grid+depth	F	Right	5	49	44	Left	Left	Dysplastic left IFG	Left IFG	Inoperable	N/a
18	Grid+depth	M	Right	12	32	20	Right	Bilateral	Right parietal dysplasia	Right SPL	FCD iib	1 (54)
19	Grid+depth	M	Right	14	49	35	Left	Left	Resected left MFG	Left SFG and MFG	DNET	3 (19)
20	Depth	M	Right	13	30	17	Left	Left	Non lesional	Left anterior medial frontal	Non-specific	5 (12)
21	Depth	M	Right	15	29	14	Left	Left	Non lesional	Left insula	Inoperable	N/a
22	Depth	M	Right	25	46	21	Right	Bilateral	Non lesional	Right orbito-/inferior frontal	Inoperable	N/a
23	Depth	M	Right	12	30	18	Right	Bilateral	Non lesional	Right mesiofrontal	Awaiting	N/a
24	Depth	M	Right	10	33	23	Right	Left	Non lesional	Right insular	Awaiting	N/a
25	Depth	F	Right	13	46	33	Left	Left	Left frontal dysplasia	Left middle frontal gyrus	Awaiting	N/a
26	Depth	M	Right	7	32	25	Left	Bilateral	Left HS	Left temporal	Awaiting	N/a
27	Depth	M	Equal	1	37	36	Left	Left	Left HS	Left hippocampus	HS	1 (4)
28	Depth	M	Right	23	27	4	Right	Left	None	Right SFG and MFG	Awaiting	N/a
29	Depth	M	Right	5	19	14	Right	Unclear	None	Right SFG	FCD iib	3 (13)
30	Depth	M	Right	1	26	25	Both	Left	None	Right SFG and MFG	Inoperable	N/a
31	Depth	F	Right	6	22	16	Left	N/a	None	Left anterior insula	Inoperable	N/a
32	Depth	M	Right	7	41	34	Left	Left	None	Left insula	Inoperable	N/a
33	Depth	M	Equal	3	44	41	Left	Right	Left frontal dysplasia	Left frontopolar/orbital	Awaiting	N/a
34	Depth	M	Right	11	41	30	Right	Left	Lesion SFG	Right SFG	Inoperable	4 (15)
35	Depth	F	Right	3	34	31	Left	Left	Left frontal dysplasia	Frontal lobe	Inoperable	N/a
36	Depth	M	Right	16	32	16	Right	Left	None	Right orbitofrontal	Gliosis	1 (36)
37	Depth	M	Right	38	8	30	Right	Left	None	Right SMA	Non-specific	1 (60)
38	Depth	M	Left	6	46	40	Right	Left	None	Right orbitofrontal	Normal	1 (6)

Abbreviations: F female, M male, ICR intracranial recording, FCD focal cortical dysplasia, SMA supplementary motor area, HS hippocampal sclerosis, DNET dysembryoplastic neuroepithelial tumour.
